# Antiphospholipid Antibodies in Women Undergoing In Vitro Fertilization Treatment: Clinical Value of IgA Anti-**β**2glycoprotein I Antibodies Determination

**DOI:** 10.1155/2014/314704

**Published:** 2014-05-25

**Authors:** Odile Paulmyer-Lacroix, Laura Despierres, Blandine Courbiere, Nathalie Bardin

**Affiliations:** ^1^Laboratoire d'Assistance Médicale à la Procréation, Hôpital de la Conception, 147 boulevard Baille, 13005 Marseille, France; ^2^Laboratoire d'Histologie-Embryologie, UFR Médecine, Université Aix-Marseille, 127 boulevard Jean Moulin, Marseille, France; ^3^Laboratoire d'Immunologie, Hôpital de la Conception, 147 boulevard Baille, 13005 Marseille, France; ^4^Department of Gynecology, Obstetric and Reproductive Medicine, Gynepole, AP-HM La Conception University Hospital, 147 bd Baille, 13005 Marseille, France; ^5^Aix Marseille Université, CNRS, IRD, Avignon Université, IMBE UMR 7263, 13397 Marseille, France; ^6^FR CNRS 3098, ECCOREV, 13100 Aix-en-Provence, France; ^7^INSERM UMRS 1076, UFR Pharmacie, Université Aix-Marseille, 127 boulevard Jean Moulin, 13005 Marseille, France

## Abstract

Implantation failure could be related to antiphospholipid antibodies (aPL). We retrospectively analyzed the usefulness of aPL determination in women undergoing IVF. Conventional aPL of the antiphospholipid syndrome, lupus anticoagulant (LA), anticardiolipin antibodies (aCL), anti-**β**2glycoprotein I (a**β**2GPI) antibodies, and IgG and IgM isotypes as well as IgA isotype were analyzed in women presenting with at least two implantation failures after in vitro fertilization (IVF). In a population of 40 IVF patients, a total prevalence of 20% (8/40) of aPL was found, significantly different from that of the control population (100 healthy blood donors, *P* < 0.0005). Among the panels of aPL tested, a**β**2GPI IgA antibodies were the most prevalent (62.5% 5/8), significantly higher in IVF patients (12.5%, 5/40) than in controls (1%, 1/100) (*P* = 0.01). No difference according to the numbers of IVF attempts and success of embryo implantation was found between aPL positive and negative IVF patients. In contrast, no accomplished pregnancy with full-term live birth was observed in aPL positive IVF patients. Altogether our data led us to propose aPL assessment, in particular a**β**2GPI IgA antibodies, in support of IVF treated women. In a perspective way, an early aPL detection could be the basis for defining novel therapeutic strategy.

## 1. Introduction

Infertility is defined as the inability to conceive after 12 months of unprotected sex and represents about 10% of the couples of childbearing age [1]. The most frequent factors of infertility are ovulation disorders, tubal obstruction, male infertility, and unexplained infertility also called idiopathic causes.

Autoimmune diseases are not considered as a major cause of infertility and their explorations are usually neglected although many of them are associated with infertility and fetal loss (defined as the loss of two by American Fertility Society or three pregnancies for others), as is the case of antiphospholipid syndrome (APS) [1, 2]. Obstetric complications are at the forefront of the symptoms of antiphospholipid syndrome. A nonimplantation could be related to unexplained infertility [3]. There are some similarities between unexplained infertility and recurrent miscarriages in APS such as defective embryonic implantation and the presence of antiphospholipid antibody (aPL) [3]. Therefore a cause of implantation failure could be related to the presence of an aPL or the existence of an APS.

In addition to the conventional markers for classification of APS, lupus anticoagulant (LA), anticardiolipin antibodies (aCL), and anti-*β*2glycoprotein I (a*β*2GPI) antibodies with IgG and/or IgM isotypes and according to the last international consensus guidelines on antiphospholipid antibodies, testing for IgA isotype is recommended for both aCL and a*β*2GPI when results of conventional markers are negative and APS is still suspected [4].

However the usefulness of aPL determination in women undergoing IVF treatment is not established. We conducted a retrospective study to assess the usefulness of aPL in laboratory testing of women undergoing IVF. To this aim, the conventional aPL of APS-LA, aCL, a*β*2GPI, IgG, and IgM isotypes as well as IgA isotype were analyzed. Finally, the relationship between the presence of aPL and embryo implantation as well as pregnancy issue was studied.

## 2. Materials and Methods

### 2.1. Patients

We retrospectively studied aPL results in women undergoing IVF attempts from 2005 to 2011. The aPL assessment was offered in our center to women when no pregnancy occurred after at least two IVF attempts with good quality embryos available for the transfer (even cleavage, even cell sizes, <20% fragmentation).

IVF treatment was performed at the Reproductive Department of University Hospital La Conception and was proposed to couples in female (ovulation disorders, tubal obstruction, and endometriosis), male, mixed, or unexplained infertility. All samples were from a declared Biobank (DC 2012-1704) with respect to ethical directives.

The control population is composed of healthy blood donors (*n* = 100).

### 2.2. Methods

#### 2.2.1. Lupus Anticoagulant Detection

For LA detection, venipuncture was performed on 0.129 M sodium citrate tubes (Vacutainer, Becton Dickinson) and platelet-poor plasma was prepared by Q1 centrifugation (twice at 3,000 g for 10 min). Whole blood and plasma fractions were stored at −80°C until use. Plasma samples were tested as recommended by the scientific and standardization subcommittee for LA of the ISTH.13 Screening for LA included activated partial thromboplastin time (aPTT, performed with LA sensitive reagent), dilute prothrombin time (1/500 dilution of thromboplastin in CaCl_2_), and dilute Russell's viper venom time (dRVVT). In the case of prolongation of screening test(s), mixing studies were performed on 1 : 1 dilution of patient plasma with pooled normal plasma. Confirmation step consisted of a dRVVT-based test with and without addition of exogenous phospholipids. In some patients, associated coagulopathies were investigated by the measurement of coagulation factors on serial dilutions of patient plasma when necessary.

#### 2.2.2. Determination of Antiphospholipid Antibodies

In-house enzyme-linked immunosorbent assays (ELISAs) were used for the determination of aCL (IgM, IgG, and IgA) and a*β*2GPI IgA, as previously described [5, 6]. Determination of a*β*2GPI (IgM and IgG) was obtained by using Orgentec ELISA kit.

In each ELISA, each sample was tested in three wells, two with antigen and one without. The latter corresponding to sample background was subtracted from the specific binding. One negative and two positive controls were included in each run. Positive controls were from patients with antiphospholipid syndrome and negative control from a healthy blood donor. For each aPL-ELISA, the cutoff level was determined by the analysis of the samples of 100 blood donors (control group) and was calculated at the 99th percentile. The results were expressed in GPLU and MPLU for IgG and IgM-aCL and a*β*2GPI, in delta optical density, for IgA isotype of aCL and a*β*2GPI. The cutoff values were the following: 1/aCL-ELISA : IgG = 20 GPLU; IgM = 8 MPLU; IgA = 0.25; 2/a2GPI-ELISA : IgG = 8 B2GU; IgM = 8 B2MU; IgA = 0.26 (GPLU, MPLU, and B2GU are arbitrary unit for, respectively, IgG IgM phospholipids and IgG *β*2glycoprotein I antibodies).

#### 2.2.3. Statistical Analysis

The results are expressed as mean ± standard deviation or percentage prevalences. The comparison of quantitative data was performed by* t*-test. The *χ* ^2^ test was used for qualitative data. A *P* < 0.05 was considered significant.

## 3. Results

A total of 40 patients could be included in an IVF program when no pregnancy was obtained after at least two embryo transfers with good quality embryos available for transfers and were examined for the presence of antiphospholipid antibody. Consequently, the studied population represented a small part of the whole population attempting IVF in our center. Mean women's age was 35 ± 4.15 years at the time of aPL detection. IVF indications were distributed as follows: female infertility (8 patients), male infertility (21 patients), mixed infertility (10 patients), and unexplained infertility (1 patient). Before starting IVF treatment, 8 women already became pregnant spontaneously (14 early miscarriages) inside the actual couple or not. At the time of aPL detection, mean number of IVF attempts was 3.85 ± 1.5 (ranging between 2 and 6) and aPL assessment was proposed only in cases where we could exclude a poor embryo quality, which is a major factor of implantation failure. Embryo transfers were performed 48 to 72 h after oocyte retrieval and the mean number of transferred embryos/transfer was 1.96 ± 0.22 (ranging between 1 and 3). At the end of the IVF program, 21 pregnancies occurred (15 patients), with 10 early miscarriages, 2 ectopic pregnancies, 6 normal deliveries, 1 premature delivery in preeclampsia context, and 2 fetal deaths in utero. We can note that one fetal death in utero occurred after the fourth IVF attempt because of venous thrombosis of umbilical cord, and the second one occurred in severe preeclampsia context within the same patient who previously had a premature delivery also in preeclampsia context. For the 8 patients presenting with secondary infertility (mean age: 35 years ± 4 ans, mean number of IVF attempts: 3.5 ± 1.2), no pregnancy was obtained after IVF.

Conventional aPL as well as aCL and a*β*2GPI of IgA isotype was analyzed in this cohort of patients undergoing IVF. A total prevalence of 20% (8/40) of aPL was found, significantly different from that of the control population (*P* < 0.0005). Among the panels of aPL tested a*β*2GPI IgA antibodies were the most prevalent (5/8, 62.5%), with a prevalence significantly higher in patients (12.5%, 5/40) than in controls (1%, 1/100) (*P* = 0.01). No significant difference was found for LA, aCL (IgG, IgM, and IgA), or a*β*2GPI (IgG and IgM). No difference was found between aPL positive and negative patients, according to history of spontaneous pregnancies before IVF treatment, the numbers of IVF attempts, and success of embryo implantation after IVF (Table 1). In contrast no accomplished pregnancy was evidenced in the aPL positive patients (Table 1). Positivity of aPL was significantly associated with abnormal IVF pregnancy outcome such as premature embryo or fetal losses. Therefore, the percent of full-term live birth after embryo implantation was significantly lower in aPL positive compared to aPL negative patients. A detailed analysis of aPL + patients presented in Table 2 evidenced that pregnancy outcome was abnormal in every case of our series (early miscarriages, fetal death in utero, or premature delivery in preeclampsia context). Within the subgroup of patients with secondary infertility, three patients were positive for aPL (Table 2). Importantly, 3 of the 5 positive women for a*β*2GP1 IgA antibodies became pregnant after IVF but without any normal delivery since one early miscarriage was evidenced for the first patient, one premature delivery and one fetal death in preeclampsia context were evidenced for the second one, and one fetal death because of venous thrombosis of umbilical cord was evidenced for the third one.

## 4. Discussion

In this study, we showed a significant higher prevalence of aPL, in particular a*β*2GPI IgA antibodies, in women undergoing in vitro fertilization treatment compared to controls. One should note that the studied population was not representative with the whole population, because patients were previously selected after at least two IVF attempts with good quality embryos available for transfers but not followed by pregnancy (implantation failure). Comparison between positive and negative aPL patients revealed no difference in success of embryo implantation, as shown by the outcome of IVF. This result was also obtained in the subgroup of patients with secondary infertility. In contrast, no accomplished pregnancy with full-term live birth was observed in aPL positive IVF patients. Altogether results led us to propose the assessment of aPL, in particular a*β*2GPI IgA antibodies, in support of IVF treated women.

Antiphospholipid antibodies (aPL) are a family of autoantibodies that are associated with pregnancy complications including stillbirth and recurrent miscarriage [7, 8]. According to data from the literature, the prevalence of aPL in infertile women varies from 4% to 66% [9]. These differences can be explained by the lack of assay standardization, the panel of antibodies tested, and the definition of population of patients. In our study, as the sample size is perhaps not representative of all the patients having a treatment of IVF, we find a total prevalence of 20%, agreeing with the average of combining results of the literature [10]. Our study highlights the high prevalence of a*β*2GPI IgA antibodies in the population of women undergoing IVF. Beta 2 glycoprotein 1, the major antigen in the antiphospholipid syndrome, is synthesized by hepatocytes, endothelial cells, and also trophoblast cells. It has been shown that this protein can adapt to different conformations, a circular, an S-shaped, and a J-shaped conformation [11]. Several studies have showed that a*β*2GPI antibodies may represent the main pathogenic antibodies in obstetrical APS [12] that could cause thrombosis of placental blood vessels, dysfunctions of trophoblasts in the peri-implantation period, or an imbalance of maternal hormones [7, 13]. In particular, Chamley et al. have proposed the action of a*β*2GPI on trophoblast proliferation as a mechanism of fetal death [14]. In addition, other studies underlined the necessity to search IgA antibodies for *β*2GPI in women undergoing IVF [6, 15]. Recently, we emphasize the determination of a*β*2GPI IgA in patients with clinical manifestations of antiphospholipid syndrome [16]. This was corroborated by Murthy et al. that recommended testing a*β*2GPI IgA when the other aPL are negative [17].

Although a larger cohort of patients should be tested to comfort our data, we did not find association between the presence of aPL and embryo implantation failure. In contrast we found a significant association between aPL positivity and abnormal pregnancy outcome as no accomplished pregnancy was obtained in aPL positive patients. These IVF treated patients could not be classified as APS patients because of their incomplete clinical expression and/or their positivity for “nonconventional aPL” (IgA isotype). Nevertheless, our results suggest that they should be considered as APS patients and thus cared for and treated accordingly. The presence of aPL and in particular a*β*2GPI IgA antibodies does not explain implantation failure but should be regarded as a pejorative factor for successful ongoing pregnancy.

## 5. Conclusions

There are various causes of reproductive failure and more than 10% are still unexplained. Testing aPL and in particular a*β*2GPI IgA should be important to exclude thrombotic state and autoimmune disease and to inform clinical practitioners. In a perspective way, it could be the basis for defining novel therapeutic strategy.

## Figures and Tables

**Table 1:** IVF cycles and pregnancy outcome in aPL positive and negative IVF treated patients.

IVF treated patients (*n* = 40)	aPL positive patients (*n* = 8)	aPL negative patients (*n* = 32)	*P* value
Patients with secondary infertility (%) (before IVF attempts)	37.5% (3/8)	15.6% (5/32)	>0.05

Mean number of IVF attempts/patient	3.75 ± 0.45	3.85 ± 0.28	>0.05
Mean number of pregnancies after IVF/patient	0.5 ± 0.7 (4/8)	0.53 ± 0.75 (17/32)	>0.05

*Outcome of IVF pregnancies *			
Abnormal outcome			
Ectopic pregnancies/pregnancy	0%	11.7% (2/17)*	<0.05*
Early miscarriages/pregnancy	25% (1/4)	52.9% (9/17)*	
Fetal death in utero or premature delivery (preeclampsia)/pregnancy	75% (3/4)	0%*	
Normal outcome			
Full-term live birth/pregnancy	0%	35.3% (6/17)	

**Table 2:** Description of aPL positive patients (*n* = 8) in IVF treated population.

Women's age (years)	IVF indication	*N* IVF attempts	*N* pregnancies	Pregnancy outcome	aPL	a*β*2GPI IgA	aCL IgG
30	Unexplained infertility	3	0		+	−	−

31	Mixed infertility	3	0		+	−	−

39	Unilateral tubal obstruction	4	3 (sp)	Early miscarriages	+	+	−

33	Female infertility	2	2 (sp)	Early miscarriages	**+**	**+**	−

30	Mixed infertility	2	1 (IVF)	Early miscarriage	**+**	**+**	−

36	Mixed infertility	6	2 (IVF)	1 premature delivery and 1 fetal death in utero (preeclampsia during these 2 pregnancies)	**+**	**+**	−

37	Male infertility	5	1 (IVF)	Fetal death in utero (venous thrombosis of umbilical cord)	**+**	**+**	−

33	Mixed infertility	4	1 (sp)	Early miscarriage	+	−	**+**

sp: spontaneous pregnancy, IVF: pregnancy after IVF cycle.
